# Urolithin A Protects against Hypoxia-Induced Pulmonary Hypertension by Inhibiting Pulmonary Arterial Smooth Muscle Cell Pyroptosis via AMPK/NF-κB/NLRP3 Signaling

**DOI:** 10.3390/ijms25158246

**Published:** 2024-07-28

**Authors:** Xinjie He, Zhinan Wu, Jinyao Jiang, Wenyi Xu, Ancai Yuan, Fei Liao, Song Ding, Jun Pu

**Affiliations:** 1 Department of Cardiology, Ren Ji Hospital, Shanghai Jiao Tong University School of Medicine, Shanghai 200127, China; urnothxj@sjtu.edu.cn (X.H.); wzn1996@alumni.sjtu.edu.cn (Z.W.); jiangjinyao@renji.com (J.J.); xuwenyi32@sjtu.edu.cn (W.X.); yuanancai@renji.com (A.Y.); liaofei@alumni.sjtu.edu.cn (F.L.); 2 Department of Cardiology, Punan Branch of Renji Hospital, Shanghai Jiao Tong University School of Medicine, Shanghai 200125, China

**Keywords:** pulmonary hypertension, Urolithin A, pulmonary smooth muscle cells, pyroptosis, AMPK

## Abstract

Recent studies confirmed that pyroptosis is involved in the progression of pulmonary hypertension (PH), which could promote pulmonary artery remodeling. Urolithin A (UA), an intestinal flora metabolite of ellagitannins (ETs) and ellagic acid (EA), has been proven to possess inhibitory effects on pyroptosis under various pathological conditions. However, its role on PH remained undetermined. To investigate the potential of UA in mitigating PH, mice were exposed to hypoxia (10% oxygen, 4 weeks) to induce PH, with or without UA treatment. Moreover, in vitro experiments were carried out to further uncover the underlying mechanisms. The in vivo treatment of UA suppressed the progression of PH via alleviating pulmonary remodeling. Pyroptosis-related genes were markedly upregulated in mice models of PH and reversed after the administration of UA. In accordance with that, UA treatment significantly inhibited hypoxia-induced pulmonary arterial smooth muscle cell (PASMC) pyroptosis via the AMPK/NF-κB/NLRP3 pathway. Our results revealed that UA treatment effectively mitigated PH progression through inhibiting PASMC pyroptosis, which represents an innovative therapeutic approach for PH.

## 1. Introduction

Pulmonary hypertension is a life-threatening disease characterized by the progressive remodeling of pulmonary arterioles, which leads to increased pulmonary artery pressure and rising pulmonary vascular resistance [1]. Consequently, the sustained PH leads to right ventricle (RV) hypertrophy and ultimately right heart failure [2]. The World Health Organization categorizes PH into five subtypes based on the underlying etiology: group 1, known as pulmonary arterial hypertension (PAH); group 2, which is PH caused by left heart disease; group 3, which is PH due to lung diseases or hypoxia; group 4, known as chronic thromboembolic pulmonary hypertension (CTEPH); and group 5, which is PH with unclear or multifactorial mechanisms [3,4]. The third subtype (group 3 PH) is the second most lethal category of PH. It is characterized by persistent pulmonary vasoconstriction, remodeling, and inflammation [5]. Although there have been notable improvements in PH treatment, the morbidity and mortality associated with PH are still increasing, and the prognosis for patients with PH remains bleak [1,6]. The primary strategy of treating PH is to expand blood vessels and reduce pulmonary vascular tension [7]. However, it is difficult for patients of group 3 PH to benefit from the existing treatments [8]. Hence, it is of great significance to elucidate the underlying mechanisms of group 3 PH and find novel therapeutic targets.

Recent studies have demonstrated that PASMC pyroptosis contributes to the pathogenesis of PH [9,10]. Pyroptosis is a form of inflammatory cell death characterized by the spontaneous and orderly death of cells, which is facilitated by the activation of caspases [11]. The NOD-like receptor (NLR) family pyrin domain-containing 3 (NLRP3) inflammasome is a complex consisting of NLRP3, apoptosis-associated speck-like protein containing CARD (ASC), and Caspase-1 [12]. Upon activation, the NLRP3 inflammasome triggers the activation of pro-Caspase-1, which results in the maturation of the pro-inflammatory cytokines interleukin-1β (IL-1β) and interleukin-18 (IL-18) [13]. Moreover, the activation of Caspase-1 in this process further induces the cleavage of gasdermin D (GSDMD), which leads to the formation of membrane oligomeric pores and membrane rupture, ultimately leading to cell death and the release of pro-inflammatory factors and cellular contents [14,15]. It was demonstrated that inflammation induced by pyroptosis augmented pulmonary vascular remodeling, resulting in the proliferation of pulmonary arterial cells, including PASMCs and pulmonary arterial endothelial cells [16,17].

Urolithins are bioactive metabolites produced by intestinal microbes from ellagitannins (ETs) and ellagic acid (EA), which are natural polyphenols found in various food sources including pomegranate, nuts, and berries [18]. Urolithins exhibit better bioavailability and greater ease of absorption compared to their predecessors [19]. During the process of digestion, there are four unique types of Urolithin compounds, namely Urolithin A-D (UA-UD). UA is the most bioactive and well-studied kind among the four Urolithin species, offering numerous advantages such as anti-inflammatory, anti-cancer, anti-oxidant, anti-aging, and muscle-function-protecting [20,21,22,23,24]. Previous studies indicated that UA could suppress NLRP3 inflammasome activation [25,26]. Nevertheless, the therapeutic potential of UA in PH has not been established.

In the present study, we aim to evaluate the protective effects of UA against PH and illustrate its underlying mechanism. Our study will further demonstrate the participation of PASMC pyroptosis in PH and provide novel strategies for PH treatment.

## 2. Results

### 2.1. Urolithin A Attenuates the Progression of Hypoxia-Induced PH

**Figure 1:**
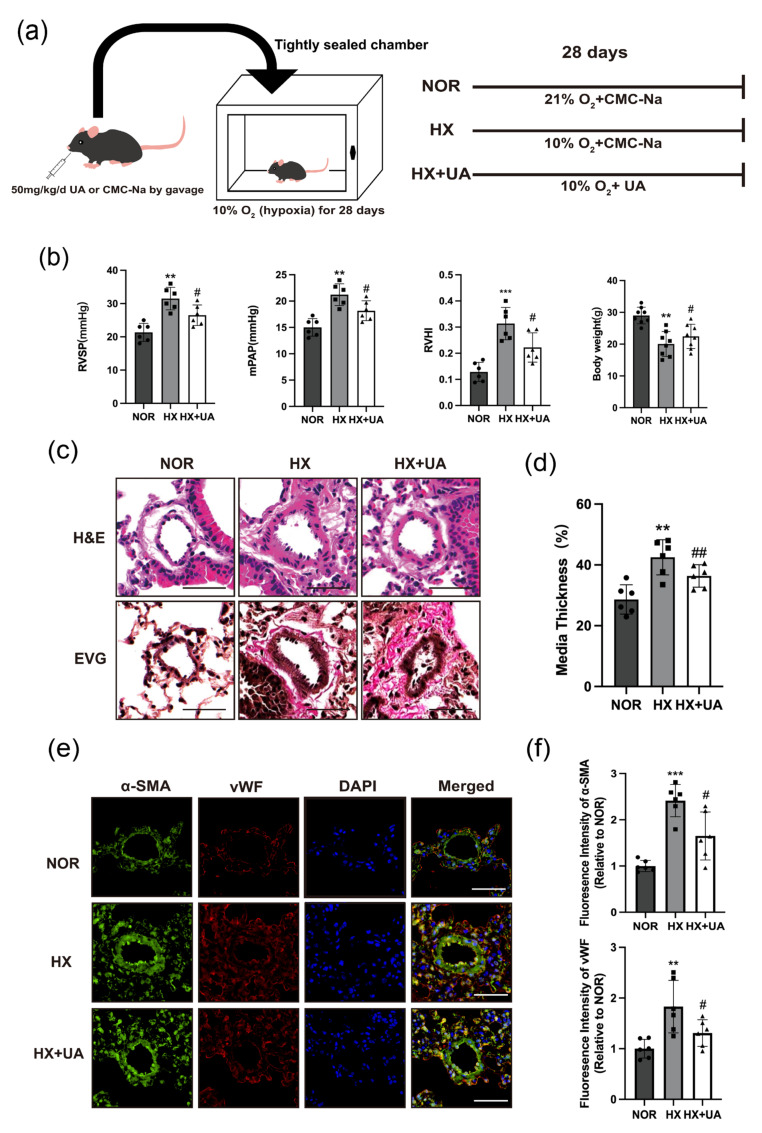
Urolithin A attenuates the progression of hypoxia-induced PH. (**a**) Protocol for administration of UA (HX + UA) or vehicle (HX) to mice subjected to hypoxia or normoxia (NOR). (**b**) RVSP, mPAP, RVHI (RVHI = RV/LV + S), and body weight in mice exposed to normoxia (NOR) or hypoxia with UA (HX + UA) or vehicle (HX) treatment (*n* = 6–8 per group). (**c**) Representative photomicrographs of hematoxylin and eosin (H&E) staining and elastin–van Gieson (EVG) staining of lung tissue from mice exposed to normoxia (NOR) or hypoxia with UA (HX + UA) or vehicle (HX) treatment (*n* = 6 per group). Scale bars: 50 μm. (**d**) Qualification of pulmonary vascular remodeling by percentage of vascular medial thickness to total vessel size for mice exposed to normoxia (NOR) or hypoxia with UA (HX + UA) or vehicle (HX) treatment (*n* = 6 per group). (**e**) Representative immunofluorescence staining of lung tissue for α-SMA (green, smooth muscle cells), vWF (red, endothelial cells) and DAPI (blue, nuclei) from mice exposed to normoxia (NOR) or hypoxia with UA (HX + UA) or vehicle (HX) treatment (*n* = 6 per group). Scale bars: 50 μm. (**f**) Qualification analysis of the α-SMA^+^ or vWF^+^ areas. ** *p* < 0.01 compared to the NOR group, *** *p* < 0.001 compared to the NOR group, # *p* < 0.05 compared to the HX group, ## *p* < 0.01 compared to the HX group.

To explore the role of UA in the development of PH, a chronic hypoxia-treated mice model of PH has been established. The hypoxia-treated mice were orally administered a 50 mg/kg dosage of UA once daily for four weeks (Figure 1a). As presented, the mice subjected to hypoxia exhibited significantly elevated RVSP, mean pulmonary arterial pressure (mPAP), RVHI, and reduced body weight compared to the normoxia group (Figure 1b). After the administration of UA, hemodynamic parameters showed considerable improvement in PH mice. Meanwhile, the mice treated with hypoxia also displayed more pulmonary arterial remodeling, which was reversed by UA intervention (Figure 1c–f). These data cumulatively highlighted that UA could prevent the development of PH by minimizing pulmonary arterial smooth muscle hyperplasia.

### 2.2. Urolithin A Inhibited NLRP3-Mediated Pyroptosis in Hypoxia-Induced PH Mice

**Figure 2:**
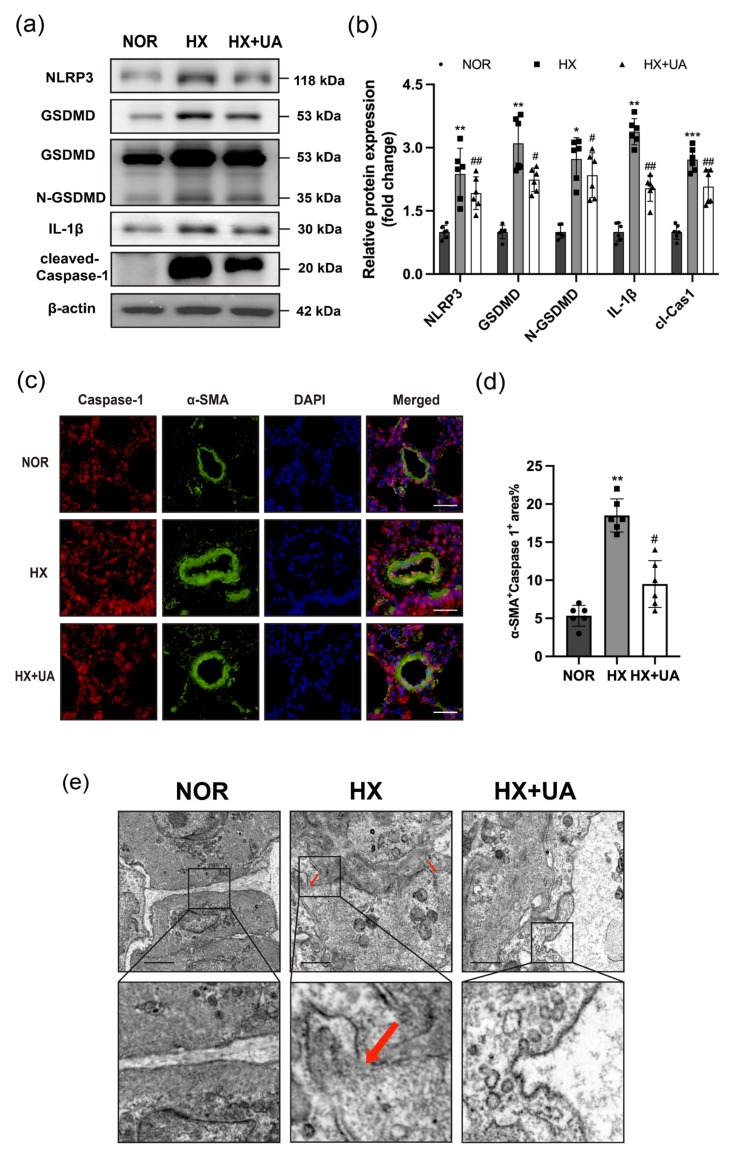
Urolithin A inhibited NLRP3-mediated pyroptosis pathway in hypoxia-induced PH mice lungs. (**a**,**b**) Western blotting analysis for the protein expression of NLRP3, GSDMD, N-GSDMD, IL-1β, and cleaved-Caspase-1 relative to β-actin from lungs of mice exposed to normoxia (NOR) or hypoxia with UA (HX + UA) or vehicle (HX) treatment (*n* = 6 per group). (**c**) Representative immunofluorescence staining of lung tissue for α-SMA (greens), Caspase-1 (red) and DAPI (blue) from mice exposed to hypoxia with UA or vehicle treatment (*n* = 6 per group). Scale bars: 50 μm. (**d**) Quantification of the α-SMA^+^ Caspase-1^+^ areas. (**e**) Representative TEM images of pulmonary arterial smooth muscle cells of mice exposed to normoxia (NOR) or hypoxia with UA (HX + UA) or vehicle (HX) treatment (*n* = 4 per group). Red arrows indicate the membrane oligomeric pores. Scale bars: 2 μm. * *p* < 0.05 compared to the NOR group, ** *p* < 0.01 compared to the NOR group, *** *p* < 0.001 compared to the NOR group, # *p* < 0.05 compared to the HX group, ## *p* < 0.01 compared to the HX group.

Subsequent examinations were conducted to assess the therapeutic impact of UA on PH in mice exposed to hypoxia. It is well known that pyroptosis plays a crucial role in the development of PH, while previous studies have pointed out that UA has the capacity to inhibit NLRP3-mediated pyroptosis [9]. Consequently, Western blotting was conducted to determine the potential influence of UA on the protein expression related to pyroptosis. As shown in Figure 2a, the protein expression level of NLRP3, cleaved-Caspase-1, and IL-1β were dramatically upregulated in the lung tissue of hypoxia-treated mice, which were subsequently reversed after UA intervention. In addition, the expression of N-GSDMD, a crucial enzyme of pyroptosis, was notably suppressed by UA (Figure 2a,b). Likewise, immunofluorescence staining results revealed an up-regulation of Caspase-1 expression in α-SMA positive cells of pulmonary artery in PH mice, which was mitigated by UA treatment (Figure 2c,d). Moreover, transmission electron microscopy (TEM) showed that the membrane of PASMCs was ruptured and cytoplasm was released after hypoxia exposure, representing the aggravation of pyroptosis, whereas it was significantly improved after UA treatment (Figure 2e). In conclusion, the above observations suggest that UA could impede the PASMC pyroptosis mediated by NLRP3 in PH mice.

### 2.3. Urolithin A Alleviated the Proliferation and Migration of hPASMCs

**Figure 3:**
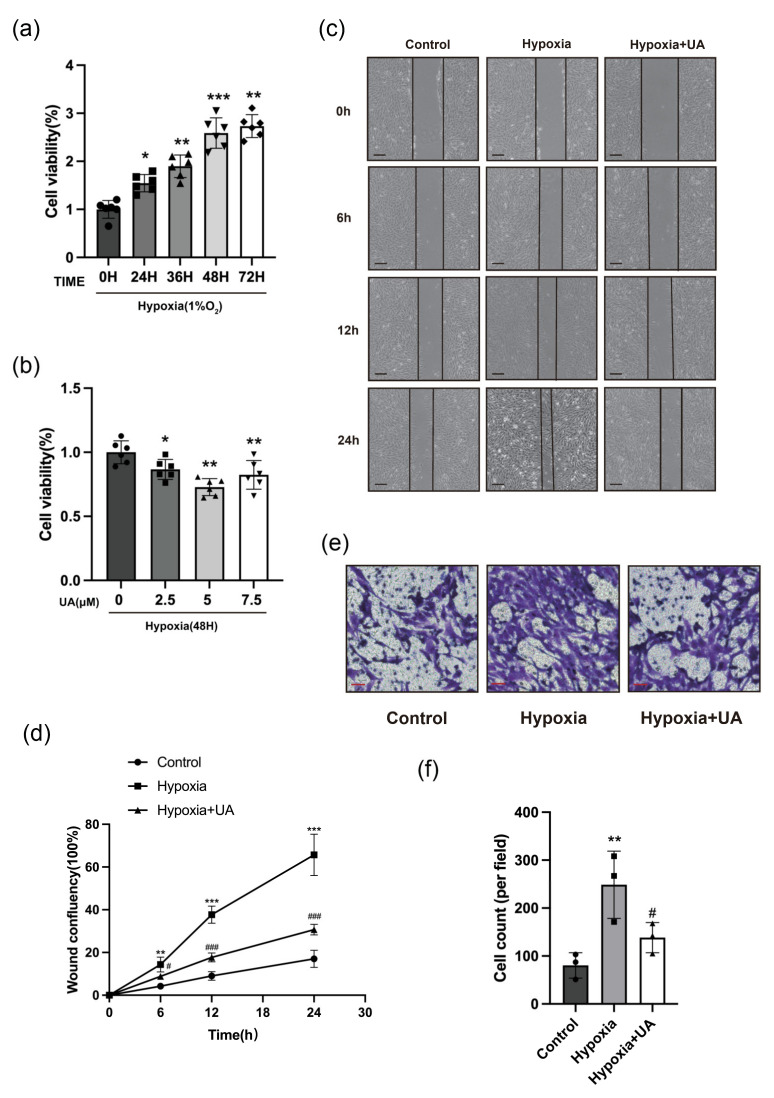
UA alleviated the proliferation and migration of hPASMCs. (**a**) Cell viability of hPASMCs exposed to hypoxia for varying durations (*n* = 6 per group). * *p* < 0.05 compared to the 0 h group, ** *p* < 0.01 compared to the 0 h group, *** *p* < 0.001 compared to the 0 h group. (**b**) Cell viability of hPASMCs exposed to hypoxia for 48 h with different concentrations of UA (*n* = 6 per group). * *p* < 0.05 compared to the 0 μM UA group, ** *p* < 0.01 compared to the 0 μM UA group. (**c**) Representative images and (**d**) qualification analysis of wound confluency of hPASMCs (*n* = 3 per group). ** *p* < 0.01 compared to the control group, *** *p* < 0.001 compared to the control group, # *p* < 0.05 compared to the hypoxia group, ### *p* < 0.001 compared to the hypoxia group. (**e**) Representative images and (**f**) qualification analysis of cell counts of hPASMCs using Transwell assay (*n* = 3 per group). Scale bars: 100 μm. ** *p* < 0.01 compared to the control group, # *p* < 0.05 compared to the hypoxia group.

PASMC proliferation is one of the most important pathological fundamentals during pulmonary vascular remodeling in PH [27]. Pyroptosis causes the release of inflammatory factors which augments the inflammatory response within vasculature. This response leads to the proliferation and migration of pulmonary arterial vascular cells, and eventually pulmonary arterial remodeling [16]. Thus, the role of UA in hPASMC (human pulmonary arterial smooth muscle cell) proliferation was evaluated after hypoxia treatment. The assessment of hPASMC proliferation was conducted using the CCK8 kit. The results showed that hypoxia facilitated the growth of hPASMCs in a time-dependent manner, in comparison with normoxia (Figure 3a). Interestingly, we observed that this alteration can be relieved by UA, with the inhibitory impact of UA being most pronounced at a concentration of 5 μM (Figure 3b). In order to assess the impact of UA administration on the migration ability of hPASMCs, a wound scratch experiment and Transwell assay were conducted. The application of UA resulted in a notable decrease in wound confluency in hPASMCs treated with UA, as compared to the control group (Figure 3c,d). Moreover, the number of hPASMCs migrating through the Transwell chamber were reduced following pre-treatment with UA (Figure 3e,f). These results indicated that UA could improve PH via protecting PASMCs against hypoxia-caused injury.

### 2.4. Urolithin A Attenuated Hypoxia-Induced Pyroptosis in PASMCs

**Figure 4:**
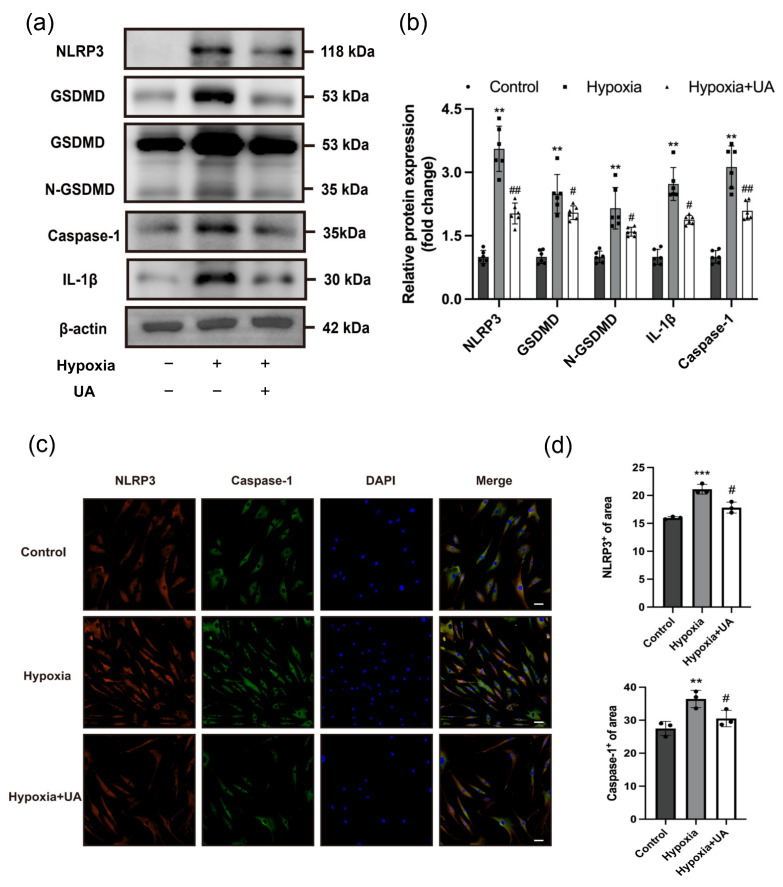
UA attenuated hypoxia-induced pyroptosis in hPASMCs. (**a**,**b**) Western blotting analysis for the protein expression of NLRP3, GSDMD, N-GSDMD, IL-1β, and Caspase-1 relative to β-actin in hPASMCs exposed to hypoxia with or without UA treatment (*n* = 6 per group). (**c**) Representative immunofluorescence staining for α-SMA (greens), Caspase-1 (red) and DAPI (blue) in hPASMCs exposed to hypoxia with or without UA treatment (*n* = 3 per group). Scale bars: 50 μm. (**d**) Qualification analysis of the NLRP3^+^ or Caspase-1^+^ areas. ** *p* < 0.01 compared to the control group, *** *p* < 0.001 compared to the control group, # *p* < 0.05 compared to the hypoxia group, ## *p* < 0.01 compared to the hypoxia group.

Various studies proved that PASMC pyroptosis contributes to the pathogenesis of PH [9,10]. To further verify the impact of UA on hypoxia-induced PASMC pyroptosis in vitro, hPASMCs were exposed to either normoxia (control) or hypoxia (1% oxygen) with or without the administration of UA (5 μM) for 48 h. Western blotting was performed to analyze the protein levels of NLRP3, GSDMD, IL-1β, and Caspase-1. The study revealed a significant increase in the expression of these proteins in PASMCs under hypoxic conditions, which was reversed by UA treatment (Figure 4a,b). Furthermore, it was observed that the fluorescence intensity of Caspase-1 and NLRP3 increased subsequent to hypoxia exposure. However, this elevation was mitigated by UA (Figure 4c,d). Collectively, these findings strongly suggested that UA could suppress the NLRP3-mediated pyroptosis pathway triggered by hypoxia in PASMCs.

### 2.5. Urolithin A Attenuated PASMC Pyroptosis through Inhibiting the NF-κB/NLRP3 Pathway

**Figure 5:**
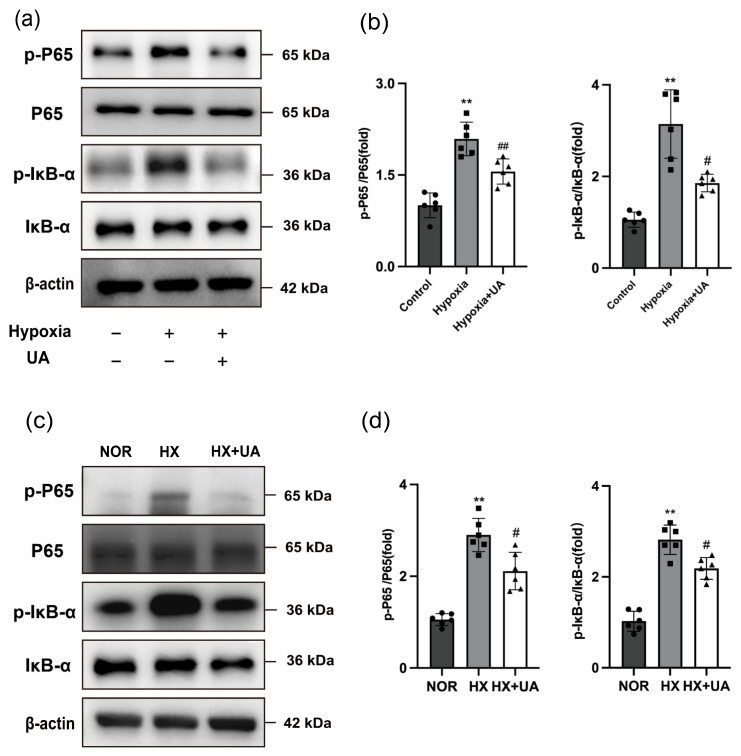
UA attenuated PASMC pyroptosis through inhibiting the NF-κB/NLRP3 signaling pathway. (**a**,**b**) Western blotting analysis for the protein expression of p-P65 relative to P65 and p-IκB-α relative to IκB-α in hPASMCs exposed to hypoxia with or without UA treatment (*n* = 6 per group). ** *p* < 0.01 compared to the control group, # *p* < 0.05 compared to the hypoxia group, ## *p* < 0.01 compared to the hypoxia group. (**c**,**d**) Western blotting analysis for the protein expression of p-P65 relative to P65 and p-IκB-α relative to IκB-α from lungs of mice exposed to normoxia (NOR) or hypoxia with UA (HX + UA) or vehicle (HX) treatment (*n* = 6 per group). ** *p* < 0.01 compared to the NOR group, # *p* < 0.05 compared to the HX group.

The NF-κB pathway is one of the key regulators of NLRP3 [28], which facilitates cellular pyroptosis and the initiation of inflammatory responses [29,30]. Multiple studies have shown the prominent involvement of the NF-κB pathway in the progression of PH [31,32]. Thus, in order to investigate the effects of UA on the NF-κB pathway, Western blotting was carried out in hypoxia-exposed hPASMCs. It was observed that the phosphorylation levels of P65 and IκB-α exhibited a significant rise following hypoxia exposure. However, these levels were effectively recovered with the administration of UA (Figure 5a,b). Similarly, in accordance with the finding in vitro, a significant improvement was observed in the NF-κB signaling pathway in hypoxia-induced PH mice treated with UA (Figure 5c,d). Altogether, our data demonstrated that UA treatment might inhibit NF-κB signaling both in vivo and in vitro.

### 2.6. Urolithin A Inhibited NF-κB/NLRP3 Pathway via Activating AMPK

**Figure 6:**
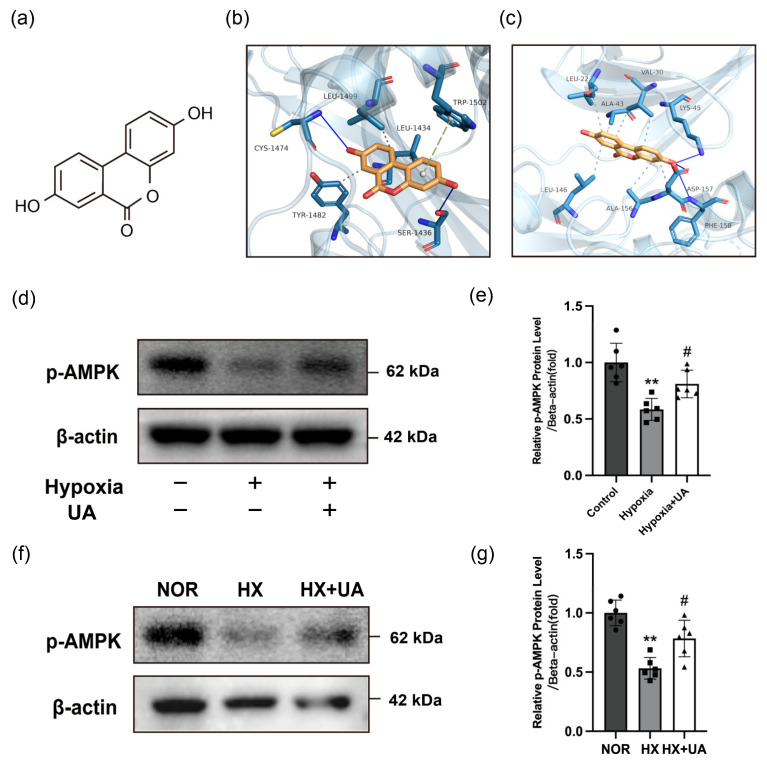
UA inhibited NF-κB/NLRP3 pathway via activating AMPK. (**a**) Molecular structure of UA. (**b**,**c**) The molecular docking models of UA with (**b**) AMPK-α1 and (**c**) AMPK-α2, respectively. The solid blue lines represent hydrogen bonds, the gray dotted lines represent hydrophobic effect, and the green dotted lines represent π-π stacking interaction. (**d**,**e**) Western blotting analysis for the protein expression of p-AMPK relative to β-actin in hPASMCs exposed to hypoxia with or without UA treatment (*n* = 6 per group). ** *p* < 0.01 compared to the control group, # *p* < 0.05 compared to the hypoxia group. (**f**,**g**) Western blotting analysis for the protein expression of p-AMPK relative to β-actin from lungs of mice exposed to normoxia (NOR) or hypoxia with UA (HX + UA) or vehicle (HX) treatment (*n* = 6 per group). ** *p* < 0.01 compared to the NOR group, # *p* < 0.05 compared to the HX group.

Previous studies have verified that UA could activate AMP-activated protein kinase (AMPK) [33,34]. In addition, AMPK is known to protect against the progression of hypoxia-induced PH [35,36,37]. Hence, we hypothesized that UA might exert a protective effect on PH by activating AMPK. To explore the interaction between UA and AMPK, a molecular docking study was conducted. As shown in the docking model, we found that UA (Figure 6a) exhibited binding affinity towards AMPK-α1 and AMPK-α2 (Figure 6b,c). In addition, the docking energy values of UA in combination with AMPK-α1 and AMPK-α2 were −8.0 and −8.4 kcal/mol, respectively. The result indicated a substantial binding affinity between UA and both AMPK-α1 and AMPK-α2. Thus, Western blotting was conducted to detect the protein expression level of AMPK. After hypoxia exposure, the expression of the phosphorylated-AMPK (p-AMPK) of hPASMCs decreased. However, this decline was successfully rescued with the administration of UA (Figure 6d,e). Consistent with the studies on hPASMCs, a notable improvement was found in the p-AMPK of hypoxia-induced PH mice administered with UA (Figure 6f,g).

**Figure 7:**
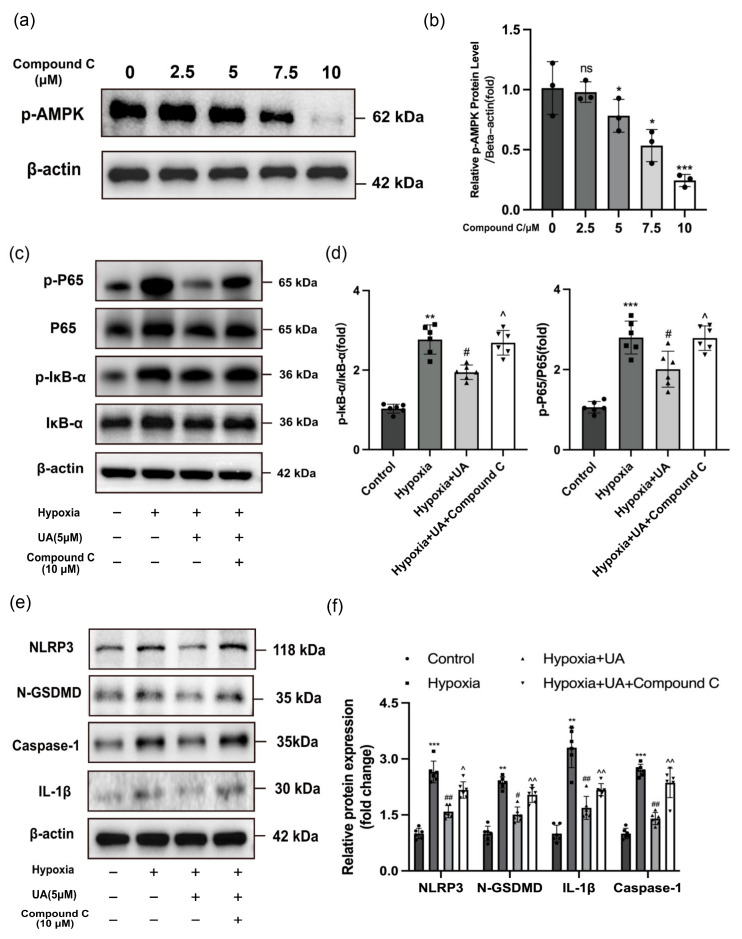
The AMPK selective inhibitor Compound C hindered the protective effect of UA on hPASMCs. (**a**,**b**) Western blotting analysis for the protein expression of p-AMPK relative to β-actin in hPASMCs administered with different concentrations of Compound C (*n* = 3 per group). * *p* < 0.05 compared to the 0μM Compound C group, *** *p* < 0.001 compared to the 0 μM Compound C group, ns means nonsignificant. (**c**,**d**) Western blotting analysis for the protein expression of p-IκB-α relative to IκB-α and p-P65 relative to P65 in hPASMCs exposed to hypoxia with or without UA or Compound C treatment (*n* = 6 per group).(**e**,**f**) Western blotting analysis for the protein expression of NLRP3, N-GSDMD, IL-1β, and Caspase-1 relative to β-actin in hPASMCs exposed to hypoxia with or without UA or Compound C treatment (*n* = 6 per group). ** *p* < 0.01 compared to the control group, *** *p* < 0.001 compared to the control group, # *p* < 0.05 compared to the hypoxia group, ## *p* < 0.01 compared to the hypoxia group, ^ *p* < 0.05 compared to the hypoxia + UA group, ^^ *p* < 0.01 compared to the hypoxia + UA group.

Next, an AMPK-specific inhibitor, Compound C, was utilized in combination with UA on hypoxia-treated hPASMCs. The Western blotting results showed a substantial decrease in the protein expression level of p-AMPK following the administration of Compound C in a dose-dependent manner (Figure 7a,b). To investigate the potential impact of Compound C on pyroptosis in hPASMCs, we also determined the expression level of pyroptosis-related proteins. The Western blotting results indicated that Compound C hindered the protective impact of UA on hPASMCs under hypoxic conditions, resulting in the elevated expression of NF-κB/NLRP3-signaling-mediated pyroptosis-related proteins p-P65, p-IκBα, NLRP3, N-GSDMD, Caspase-1, and IL-1β (Figure 7c–f).

Above all, these findings indicated that UA inhibits PASMC pyroptosis via the AMPK/NF-κB/NLRP3 pathway, therefore ameliorating the progression of hypoxia-induced PH.

## 3. Discussion

**Figure 8:**
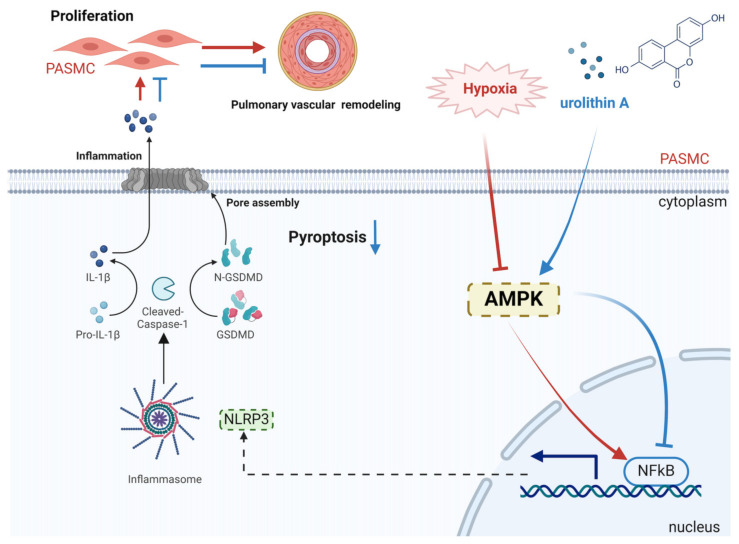
Urolithin A protects against hypoxia-induced pulmonary hypertension by inhibiting NF-κB/NLRP3-mediated PASMC pyroptosis via regulating AMPK signaling (created with BioRender.com). PASMC, pulmonary arterial smooth muscle cell; IL-1β, interleukin-1β; GSDMD, gasdermin D; NLRP3, NOD-like receptor (NLR) family pyrin domain-containing 3; AMPK, AMP-activated protein kinase.

The present study indicated that UA exerts a beneficial impact against the pathogenesis of hypoxia-induced PH via reducing PASMC pyroptosis. In addition, the mechanistic investigation revealed that UA inhibits PASMC pyroptosis via the AMPK/NF-κB/NLRP3 pathway. Collectively, our findings creatively provide a novel strategy for PH treatment (Figure 8).

Prolonged chronic hypoxia is a significant factor in the development of group 3 PH. Hypoxia is one of the prevalent triggers for cellular pyroptosis, which is a form of cellular death characterized by pro-inflammatory features, predominantly driven by Caspase-1 activation. The activation of Caspase-1 is initiated by the inflammasomes [38]. The fundamental constituent of inflammasomes, comprising pyrin, the NOD-like receptor (NLR) family (NLRP1, NLRP3, NLRP6, NLRP9, and NLRC4), and absent in melanoma 2 (AIM2) [39]. The NLRP3 inflammasome has been found to contribute to the progression of PH [40]. Bauernfeind et al. reported that the activation of the NF-κB pathway leads to the upregulation of NLRP3 inflammasome subunits, including NLRP3, ASC, and pro-Caspase-1 [41]. Upon activation of the NLRP3 inflammasome, it triggers the process of pro-Caspase-1 self-cleavage and activation, which results in the maturation of the proinflammatory cytokines IL-1β and IL-18 [12]. Simultaneously, the activation of Caspase-1 results in the cleavage of GSDMD, converting it into N-GSDMD. Following that, N-GSDMD translocates to the cell membrane, inducing the formation of membrane pores through the formation of membrane oligomerized complexes. This process leads to the leakage of cellular contents and inflammatory factors [14]. The pathophysiological process of pulmonary vascular remodeling, which is a significant feature of PH, has been proven to be associated with the inflammatory response and immune system [16,42]. Research related to pyroptosis and inflammasome has predominantly concentrated on immunity cells [43,44,45]. However, previous studies have found that PASMC pyroptosis plays a key regulatory role in the pathogenesis of PH. The release of inflammatory cytokines due to pyroptosis induces the proliferation and intense inflammatory response of PASMCs [17,30,46] and the malfunction of pulmonary endothelium cells [47], which is known to be the fundamental of PH progression.

Extensive research has been conducted in recent years to examine the preventative effects of UA on various cardiovascular diseases, including cardiomyopathy, myocardial fibrosis, atherosclerosis, and myocardial ischemia [24,48,49,50]. UA is a naturally derived byproduct of ellagitannins, which are synthesized by the gut microbiota and found in many fruits such as pomegranates or almonds [19]. The gut microbiota exhibits an influence on the regulation of PH [51]. Tao et al. indicated that UA has the capability to impede NF-κB/NLRP3-pathway-mediated pyroptosis [25]. In addition, UA possesses an anti-inflammatory effect in many diseases such as inflammatory bowel disease [52], septic cardiomyopathy [53], and atherosclerosis [54]. However, the specific role of UA on PH has yet to be investigated.

Our study demonstrated that pyroptosis contributed to PH progression by establishing hypoxia-induced PH models in vivo and in vitro. Moreover, we treated mice and PASMCs with UA under the hypoxia condition and observed that UA effectively suppressed hypoxia-induced pyroptosis via inhibiting the NF-κB/NLRP3 pathway both in vivo and in vitro. AMPK, as an essential energy sensor in cellular metabolism, plays a key role in several responses, including inflammation, neurodegeneration, and oxidative stress [55]. Previous studies have provided evidence that AMPK could be activated by UA [34]. Furthermore, the development of PH is associated with a reduction in AMPK activation [37]. Thus, we utilized Compound C, a selective AMPK inhibitor, in conjunction with UA to treat hPASMCs. We found that Compound C effectively inhibits the effect of UA via inhibiting the NF-κB/NLRP3 pathway in hypoxia-induced PASMCs. In conclusion, it has been shown that UA may have a protective impact on the pyroptosis of hypoxia-induced PASMCs through the activation of AMPK, which subsequently inhibits the NF-κB/NLRP3 pathway.

Our study has certain limitations; we only studied PASMCs in the study and verified the role of UA on PASMC pyroptosis. However, previous studies demonstrated that immunity cells play an important role in hypoxia-induced PH [56,57,58]. Therefore, whether administering UA can regulate immunity cells in group 3 PH requires further study.

Ultimately, our study has initially verified that AMPK activation could effectively suppress pyroptosis in PASMCs induced by hypoxia. Furthermore, we first confirmed that UA could impede PASMC pyroptosis in response to hypoxia and protect against PH, which suggested that UA could serve as a potential protective approach for future PH treatment.

## 4. Materials and Methods

### 4.1. Chemicals

Urolithin A (purity ≥ 98%), Dorsomorphin (Compound C, purity ≥ 99%), and sodium carboxymethyl cellulose (CMC-Na, purity ≥ 98%) were purchased from Selleckchem (Selleck Chemicals LLC., Houston, TX, USA). Dimethyl sulfoxide (DMSO, purity ≥ 99%) was purchased from Sigma Chemical Co. (Sigma-Aldrich, St. Louis, MO, USA).

### 4.2. Animal Model and Experiment

Male C57BL/6 mice (20–25 g) aged 6–8 weeks were purchased from Shanghai Research Center of Southern Model Organisms (Shanghai, China). All animal experiments strictly followed the National Institutes of Health Guidelines on the Use of Laboratory. The protocols on the ethics of animal use were approved by the Animal Ethics Committee of Ren Ji Hospital, Shanghai Jiao Tong University School of Medicine.

The mice were kept in cages with 4–5 mice in each, provided with unrestricted food and water, and housed in a standard specific-pathogen-free (SPF) laboratory conditions with a 12 h light–dark cycle and adapted to the environment for one week. UA was suspended in 0.5% CMC-Na. Specifically, the mice were randomly assigned to three groups (*n* = 10): (1) normal control group exposed to normoxia with 0.5% CMC-Na treatment; (2) model group exposed to hypoxia with 0.5% CMC-Na treatment; (3) Urolithin A group exposed to hypoxia with UA gavage at 50 mg/kg according to previous studies [24,26]. The control group of mice were exposed to normobaric normoxia (21% O_2_) in room air, while the chronic hypoxia group of mice were treated with normobaric hypoxia (10% O_2_) in a tightly sealed chamber for 4 weeks [59,60]. On day 28, all animals were sacrificed for analysis.

### 4.3. Hemodynamic Measurement

After 4 weeks, the RV systolic blood pressure (RVSP) of the mice was measured with pressure transducers under anesthesia. The mice were anesthetized with 3.0–3.5% isoflurane gas in an airtight chamber, then kept under anesthesia with 1.0–1.5% isoflurane gas using an intubation tube before being ventilated with a rodent respirator. For measuring RVSP, a 1.2-F microtip pressure transducer catheter (ADinstrument, New South Wales, Australia) was connected to a pressure transducer and inserted into the right ventricle through the right jugular vein. The right ventricular hypertrophy index (RVHI) was assessed using the ratio of right ventricular free wall weight to the sum of left ventricular and septal weight: RVHI = RV/(LV + S).

### 4.4. Transmission Electron Microscopy

The membrane oligomeric pores of PASMCs in mice pulmonary arteries were observed using a Transmission Electron Microscope (TEM). Briefly, freshly isolated mice lung tissues were fixed in a TEM fixation solution for 2 h at 4 °C. Then, the tissues were washed with 0.1 M phosphate buffer PB three times, 15 min each time, followed by post-fixation with 1% osmic acid at 4 °C for 2 h. After that, the tissues were rinsed three times, 15 min each time, and then dehydrated with a series of graded ethanol solutions. Next, the tissues were embedded in an embedding agent (90529-77-4, SPI, West Chester, PA, USA) overnight. The tissues were then cut into 70 nm ultrathin slices using a microtome (Leica UC7, Leica, Wetzlar, Germany) and stained with lead citrate and 2% uranyl acetate for 15 min each. Finally, the sections were ultimately examined by TEM (TECNAI G2 20 TWIN, FEI, Hillsboro, OR, USA).

### 4.5. Cell Culture and Treatment

The human pulmonary artery smooth muscle cells (hPASMCs) used in the cell experiments were purchased from Procell (CP-H007, Wuhan, China). The cells were cultured in smooth muscle cell growth medium and incubated at 37 °C in a humidified atmosphere of 5% CO_2_. For the hypoxia experiments, cells were placed in the modular incubator chamber (Fisher Scientific, Waltham, MA, USA) with 1% O_2_ with or without UA or Compound C for 48 h.

### 4.6. Cell Counting Kit 8 Assay

To evaluate cell proliferation, hPASMCs were seeded in 96-well plates and cultured overnight. Following treatment according to the various experimental groups, a cell counting kit 8 solution (CK04, Dojindo Laboratories, Shanghai, China) was introduced into each well and cells were incubated at 37 °C, 5% CO_2_ for 1.5 h. Then, the results were detected at a wavelength of 450 nm utilizing a NanoDrop 2000c spectrophotometer (Fisher Scientific, Waltham, MA, USA).

### 4.7. Wound Scratch Assay

HPASMCs were seeded in 6-well plates and cultivated until they reached 80% confluence. The scratches were subsequently made with a pipette tip. The cells were washed twice with PBS and were thereafter exposed to different treatments. The images were obtained at ×20 magnifications at an indicated time point post-scratching. The scratch’s original boundaries at hour 0 were marked, and the distance it had migrated at each time point was measured using an inverted microscope (Leica DMI3000 B, Leica, Wetzlar, Germany). The scratch area was measured using ImageJ 1.53k software (National Institutes of Health, Bethesda, MD, USA), and the scratch confluence was determined using the formula: scratch confluence % = (initial scratch area − scratch area at different times)/initial scratch area × 100%.

### 4.8. Transwell Assay

The Transwell experiments were conducted utilizing an 8 μm Transwell chamber (Corning Costar Corp., Corning, NY, USA). HPASMCs were cultured at a density of approximately 1 × 10^5^ cells/mL using a fresh culture medium (100 μL) devoid of FBS. Subsequently, a volume of 600 μL of complete medium was introduced into the lower chambers, and the cells were transplanted into the upper chamber and conducted with different treatments. Following incubation, we delicately moved the cells present on the top chamber’s surface, immobilized the migrating cells adhered to the lower chamber’s surface, and subjected them to staining using 0.4% crystal violet. Then, cells were washed twice with PBS, and kept dry at room temperature (RT) overnight prior to capturing images. Microscopy was employed to assess the migratory cell count in five fields that were randomly selected. Three independent experiments were performed. The ImageJ 1.53k software was utilized to quantify the cell count in every field of view.

### 4.9. Hematoxylin and Eosin (H&E) and Elastin–van Gieson (EVG) Staining

For histological analysis, the lung tissues were fixed in 4% paraformaldehyde at room temperature for 24 h. Subsequently, they were embedded in paraffin and sliced into 5 μm thick sections. The slices were stained with hematoxylin and eosin and elastin–van Gieson following the standard protocol for histological analysis and the internal and external diameter of pulmonary arteries were measured by ImageJ 1.53k software. The formula for calculating pulmonary arterial medial wall thickness (WT) percentage is as follows: WT% = (External diameter − Internal diameter)/External diameter × 100%.

### 4.10. Immunofluorescence Staining

For immunofluorescence analysis, PASMCs were seeded on coverslips in 12-well plates and incubated overnight. Following the treatment, the cells were subjected to fixation using 4% paraformaldehyde solution for 15 min. Subsequently, cells were permeabilized using Immunostaining Permeabilization Solution with Triton X-100 (Beyotime, Shanghai, China) for 10 min at RT. Subsequently, the cells were subjected to blocking using Immunol Staining Blocking Buffer (Beyotime, Shanghai, China) at RT for 10 min and treated with the primary antibodies overnight at 4 °C, followed by incubation with Alexa 594-labelled or Alexa 488-labelled secondary antibodies (Invitrogen, Carlsbad, CA, USA) in the dark at RT for 1 h. Finally, 4′,6-diamidino-2-phenylindole (DAPI) was added to visualize the nuclei. The primary antibodies used in the experiment are listed as follows: rabbit polyclonal antibody against NLRP3 (1:200, 27458-1-AP, Proteintech, Wuhan, China) and rabbit monoclonal antibody against Caspase-1 (1:200, 22915-1-AP, Proteintech, Wuhan, China).

The paraffin sections of mice lung tissues were co-incubated with primary antibodies against α-SMA (1:200, 67735-1-AP, Proteintech, Wuhan, China) and v-WF (1:200, ab287962, Abcam, Cambridge, UK) or primary antibodies against α-SMA and Caspase-1 (1:200, 22915-1-AP, Proteintech, Wuhan, China) at 4 °C overnight. Subsequently, the sections were incubated with secondary antibodies at RT for 1 h. DAPI was added to visually represent the nuclei.

Images were captured using a laser scanning confocal microscope (Leica TCS SP8, Leica, Wetzlar, Germany). All photos were quantified and analyzed using ImageJ 1.53k software obtained from an online source.

### 4.11. Protein Extraction and Western Blotting Analysis

Briefly, lung tissues and hPASMCs were lysed in RIPA lysis buffer (premixed with protease and phosphatase inhibitors) for 15 min at 4 °C to extract the total protein. Furthermore, the BCA protein assay kit (Thermo Scientific, Waltham, MA, USA) was utilized to measure the protein concentrations of protein samples according to standard protocols. Equal amounts of protein extracts (30 μg/well) were loaded onto 10% or 12.5% SDS-PAGE gels, separated by electrophoresis running at 80 V for 0.5 h and subsequently at 120 V for 1.5 h, and then transferred onto 0.45 μm polyvinylidene fluoride (PVDF) membranes (Millipore, Billerica, MA, USA). The membranes were incubated overnight at 4 °C with specific primary antibodies. The primary antibodies used in the experiment are listed as follows: rabbit monoclonal antibody against NLRP3 (1:1000, ab270449, Abcam, Cambridge, UK), IL-1β (1:1000, ab254360, Abcam, Cambridge, UK), GSDMD (1:1000, ab209845, Abcam, Cambridge, UK), Caspase-1 (1:1000, 22915-1-AP, Proteintech, Wuhan, China), Cleaved Caspase-1 (1:1000, #89332S, CST, Danvers, MA, USA), phospho-AMPK (1:1000, #2535S, CST, Danvers, MA, USA), NF-κB p65 (1:1000, AF1234, Beyotime, Shanghai, China), phospho-NF-κB p65 (1:1000, AF5875, Beyotime, Shanghai, China), IκB-α (1:1000, AF1282, Beyotime, Shanghai, China), phospho-IκB-α (1:1000, ab92700, Abcam, Cambridge, UK), β-actin (1:1000, AF5003, Beyotime, Shanghai, China), rabbit polyclonal antibody against NLRP3 (1:1000, 27458-1-AP, Proteintech, Wuhan, China), and GSDMD (1:1000, 22770-1-AP, Proteintech, Wuhan, China). The PVDF membranes were then washed with TBST three times and incubated with the secondary antibodies for 1 h and washed three times at RT. Finally, the immunoblot bands were visualized using enhanced chemiluminescence regents (Millipore, Billerica, MA, USA) with the ChemiDoc^TM^ Imaging system (Bio-Rad Laboratories, Hercules, CA, USA). The quantification of signal intensities was analyzed using an image J software.

### 4.12. Molecular Docking

A molecular docking study was conducted to investigate the interaction between UA and AMPK. First, the molecular structures of the target proteins AMPK-α1 (PDB ID: 7MYI) and AMPK-α2 (PDB ID: 8HAN) were found and downloaded from the Protein Data Bank. The Chem3D16.1 software was used to optimize the optimal conformation of target proteins, and finally, the optimal conformation with minimal energy was obtained. The molecular structure of UA was acquired from PubChem Compound. The target proteins were preprocessed using Auto Dock Tools 1.5.6 and the Auto Dock Vina was used to predict the binding model between UA and AMPK, with docking runs conducted using the Lamarckian genetic algorithm. The docking results were visualized in 3D using PyMOL2.3.0.

### 4.13. Statistical Analysis

All experiments were conducted in a blinded manner and repeated at least three times independently, and consistent results were obtained. All statistical analyses were conducted using IBM-SPSS v.24.0 (IBM). Data were presented as mean ± SD. Two independent sample data analyses were conducted using a one-sided t test. One-way analysis of variance (ANOVA) followed by Tukey’s post-hoc test was used for sets of data. Categorical variables were analyzed by chi-square analysis. *p* < 0.05 was deemed statistically significant.

## 5. Conclusions

In summary, our study demonstrated that UA effectively prevented the development of hypoxia-induced PH in mice through mitigating PASMC pyroptosis by modulating AMPK/NF-κB/NLRP3 signaling. Our findings indicated that UA may serve as a therapeutic option for patients with group 3 PH.

## Data Availability

The data in the current study are available from the corresponding author upon reasonable request.
